# Supplementary material for: Effects of (2R,6R)-hydroxynorketamine in assays of acute pain-stimulated and pain-depressed behaviors in mice
Source: PLoS One. 2024 Apr 19;19(4):e0301848. doi: 10.1371/journal.pone.0301848 (PMC11029659; doi:10.1371/journal.pone.0301848)
Supplement: S1 File — (PDF) [file pone.0301848.s001.pdf]

Certificate of analysis for (*2R,6R*)-Hydroxynorketamine hydrochloride

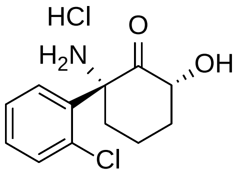

Compound name: (*2R,6R*)-2-amino-2-(2-chlorophenyl)-6-hydroxycyclohexanone hydrochloride (2R,6R-HNK, NCGC00378227)

Lot number: NCGC00378227-26

Manufacturing date:

Manufacturer: (name, address): January 3rd, 2018

Patrick Morris
National Center for Advancing Translational Sciences
9800 Medical Center Drive
Rockville, MD 20850

Analysis

Patrick Morris
National Center for Advancing Translational Sciences
9800 Medical Center Drive
Rockville, MD 20850

Appearance: White granular solid

Identity: $C_{12}H_{14}Cl_1NO_2$ (2R,6R-HNK), $C_{12}H_{15}Cl_2NO_2$ (2R,6R-HNK HCl)

^{1}H NMR, ^{13}C NMR: conforms (see attached)

MS: HRMS: *m/z* (M+H) = 240.0784 Calculated for $C_{12}H_{15}Cl_1NO_2$ = 240.0786

$[\alpha]_D^{20}$: -113.04° (*c* 1.0, H_2O).

Enantiomeric Excess: (See attached chiral LC) > 99.5 %

Purity: (HPLC): 99.5% @ 220 nM

Residual Organic Solvents: Acetone, 0.6%

C,H,N,Cl Analysis: Expected: C 52.19%, H 5.48%, N 5.07%, Cl 25.67%

Observed: C 51.84%, H 5.38%, N 4.84%, Cl 25.98 %

Karl Fisher: 0.13%

Melting Point: 248.4°C

Palladium: <1 ppm

DSC: see attached

TGA: see attached

Signature of the person that generated the COA:

Patrick J. Morris

Material is pure and correct as judges by LC-MS and NMR (see below). Long term storage at cold or ambient temperatures in the dark. As a powder, the sample can be stored for up to 24 months. In solution, the sample can be stored for 4 weeks in a cool/dark environment. Limit freeze/thaw cycles. The material is soluble in water up to 500 mg per 1 ml.

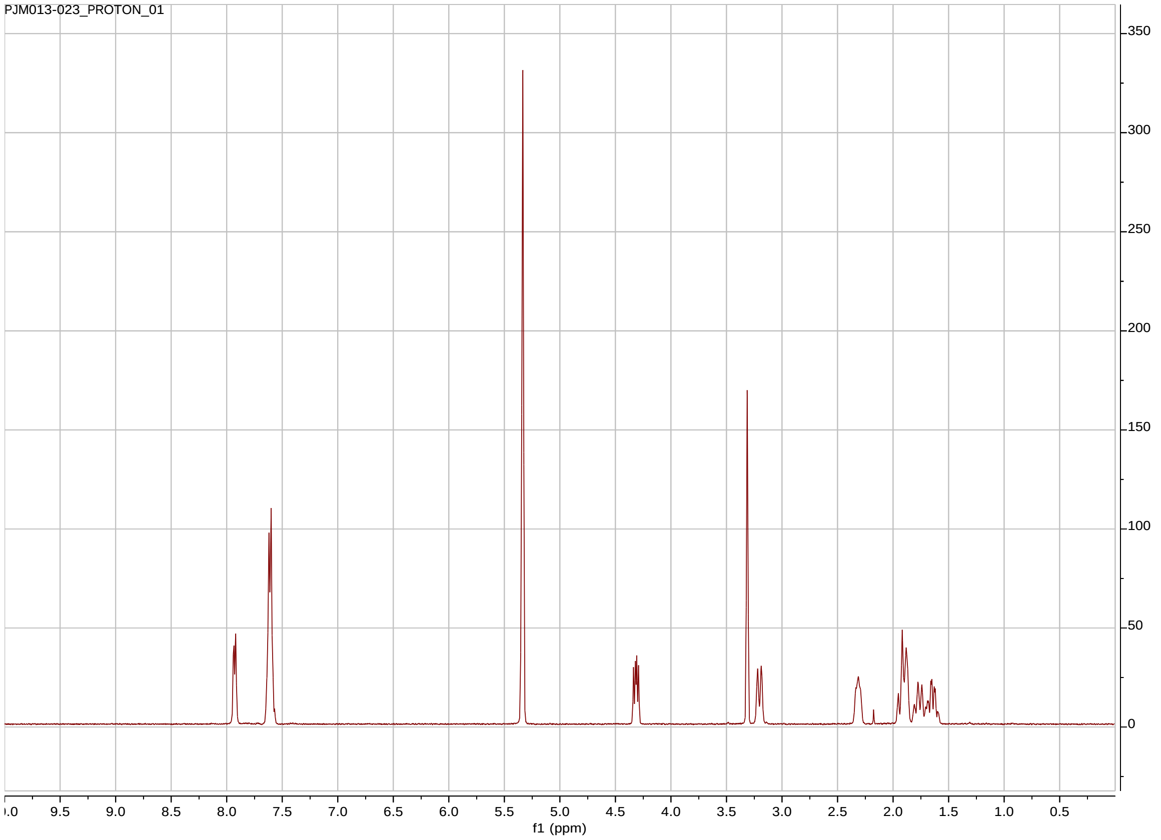

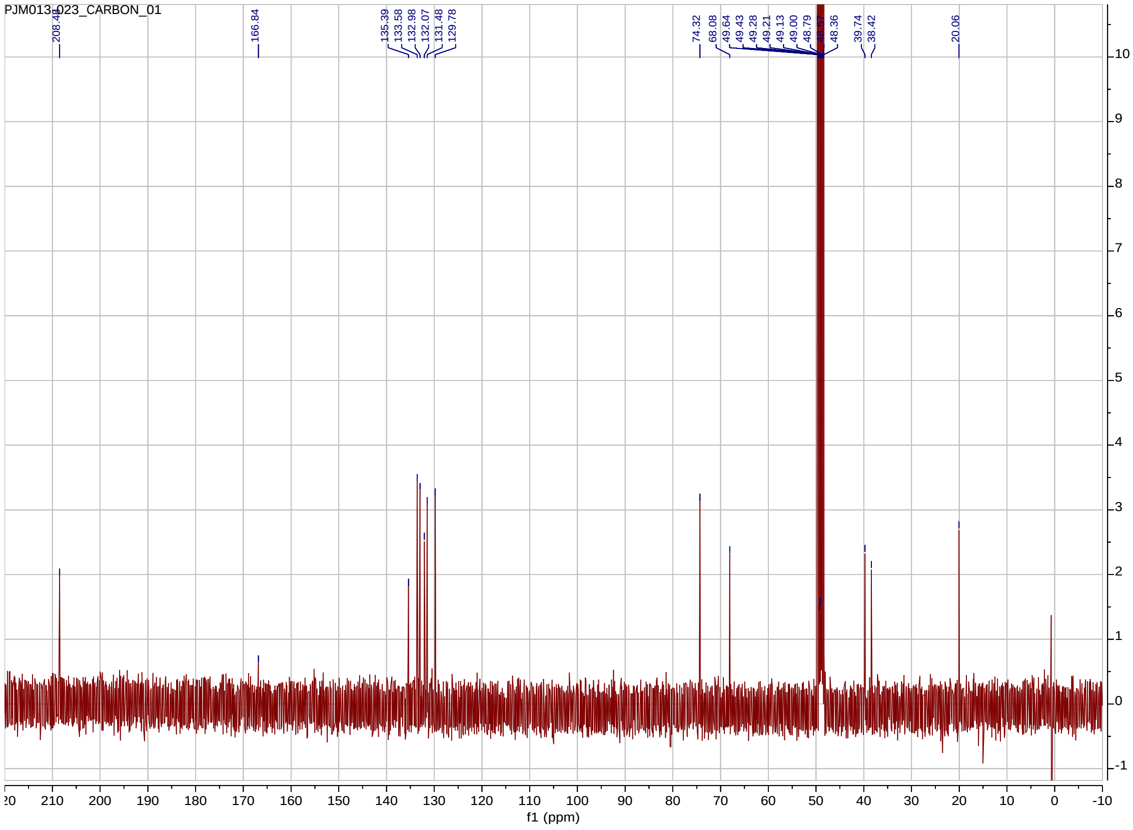

File ..1\DATA\PJM\PJM013-035 2018-01-26 12-54-15.D Tgt Mass (EZX):

Injection Date : 26-Jan-18, 12:55:25 Seq. Line : 0
Sample Name : PJM013-035 Location : Vial 1
Acq. Operator : SYSTEM Inj : 0
Spec. Reported : <None> Inj Volume : 2 ul
Acq. Method : C:\CHEM32\1\METHODS\CHIRAL METHODS\CHIRAL ANALYTICAL METHODS\AD60ETOH.M
Analysis Method : C:\CHEM32\1\METHODS\CHIRAL METHODS\CHIRAL ANALYTICAL METHODS\AD60ETOH.M
Sample Info : AD, Hex/EtOH/DEA 40:60:0.04, 2 uL, 1.0 mL/min,
Method Info : Chiralpac AD 4.6x250
60% ethanol/hex 1 mL/min

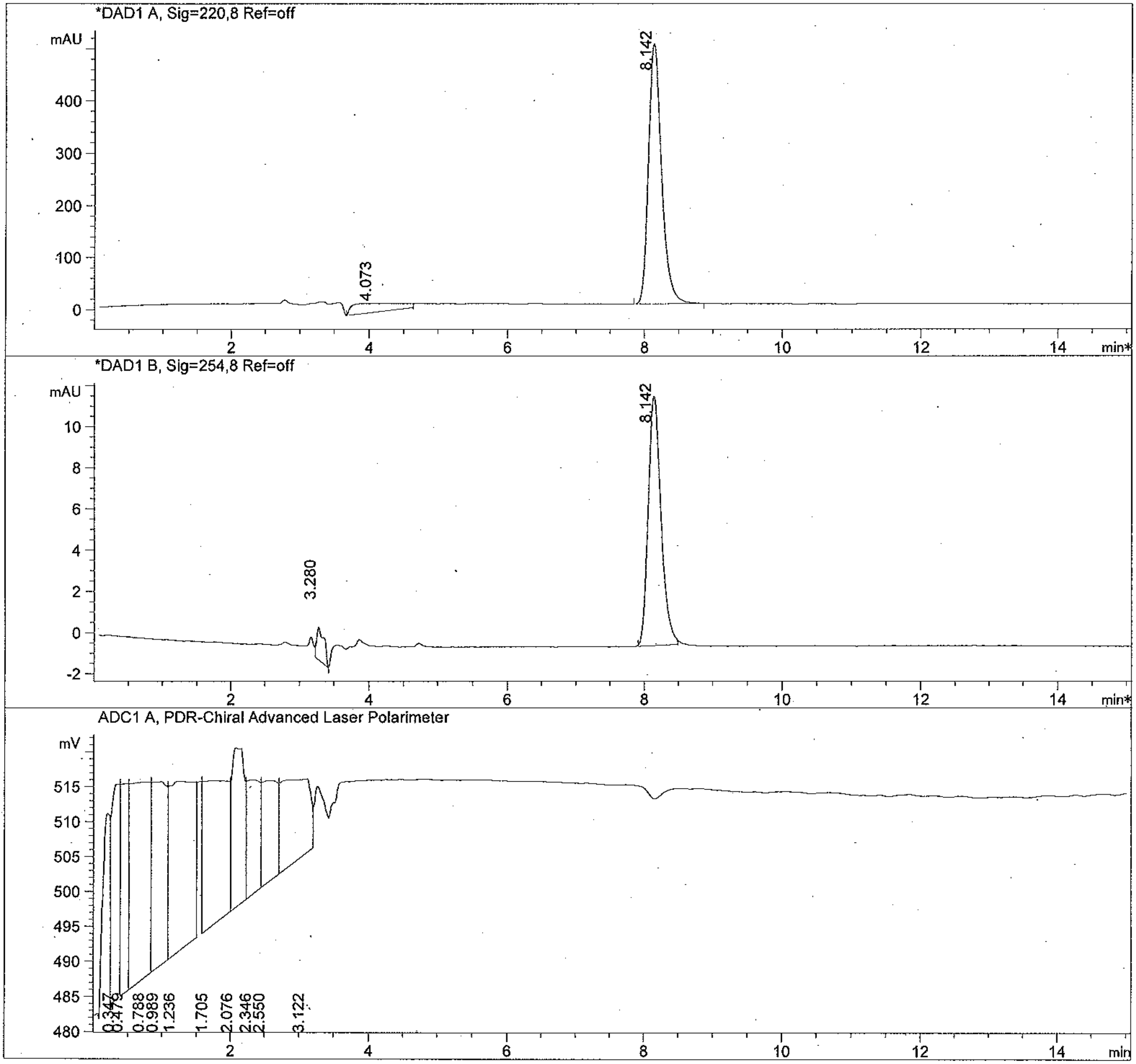

File ..W\MORRISPJ\01-18\020118-PJM013-0231-03028.D Tgt Mass (EZX):

Injection Date : 02-Jan-18, 15:13:40 Seq. Line : 0
Sample Name : pjm013-023 Location : P1-B-03
Acq. Operator : Patrick Morris Inj : 0
Spec. Reported : UV Integration Inj Volume : 3 ul
Acq. Method : C:\Chem32\1\METHODS\FINAL_GRAD_NO_PRINT.M
Analysis Method : C:\Chem32\1\METHODS\FINAL_GRAD_NO_PRINT.M
Sample Info : Easy-Access Method: 'SUBMISSION'
Method Info : Standard Gradient 4% to 100% Acetonitrile (0.05% TFA) over 7 minutes
Luna C18 3 micron 3 x 75mm

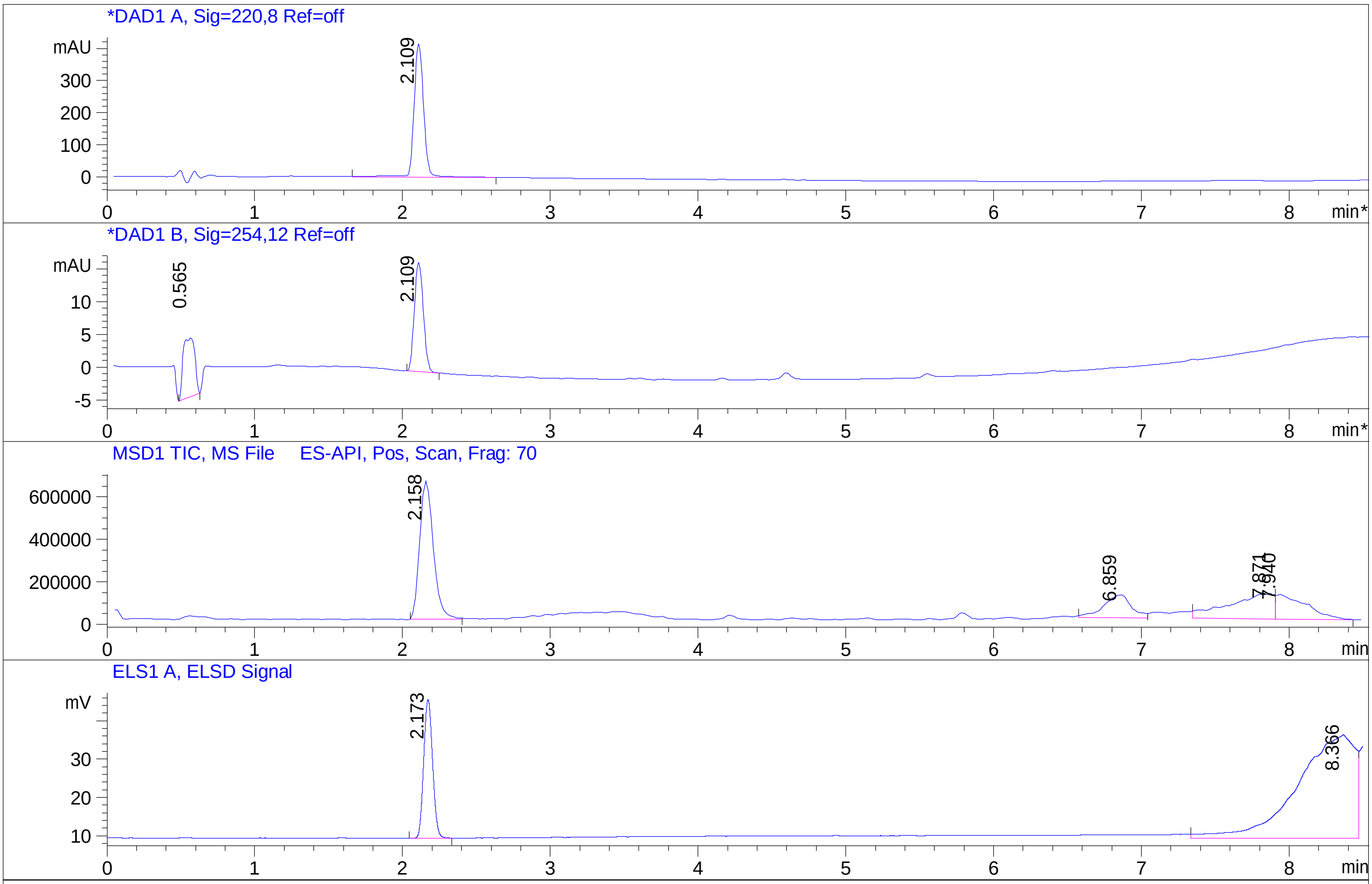

Integration Results for DAD1 A, Sig=220,8 Ref=off

RetTim	Width	Area	Height	Area%	MS(+)
2.11	0.07	1914.53	416.96	100.00	240

Integration Results for DAD1 B, Sig=254,12 Ref=off

RetTim	Width	Area	Height	Area%	MS(+)
0.57	0.08	49.80	8.96	41.25	179
2.11	0.07	70.93	16.76	58.75	240

Ret. Time: 0.57 <<<< POSITIVE SPECTRA >>>> Ret. Time: 2.11

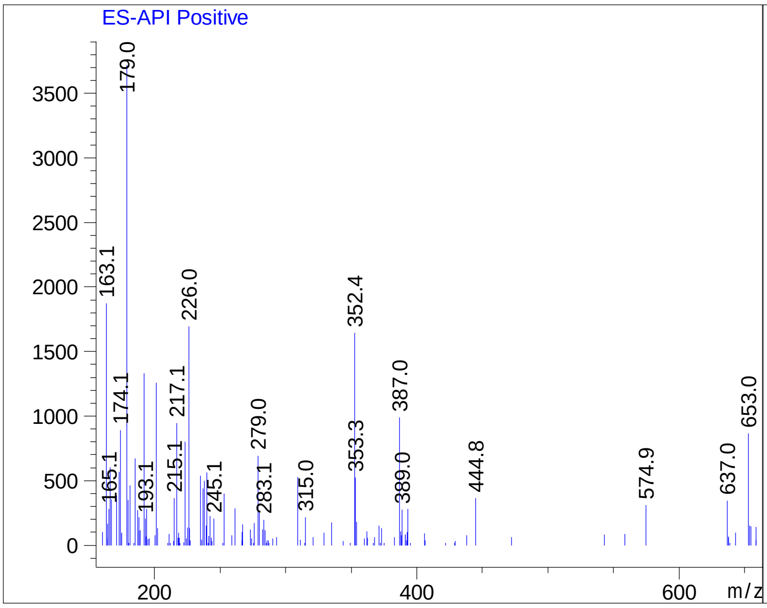

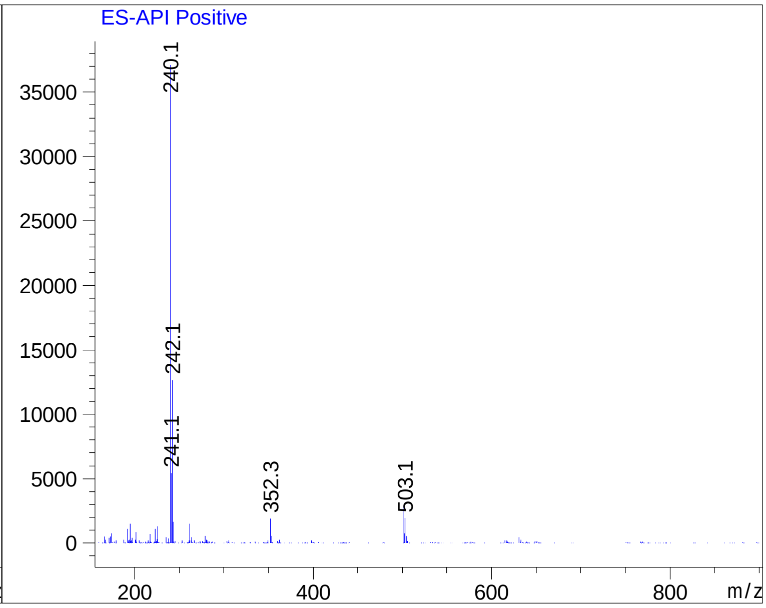

Filename:	C:\Program Files (x86)\Per...\20180049.ds8d
Operator ID:	Dipti Parikh
Sample ID:	NCGC00378227-26 : 20180049
Sample Weight:	4.340 mg
Comment:	P Morris
	1-24-18
	Sealed Pan

PerkinElmer Thermal Analysis

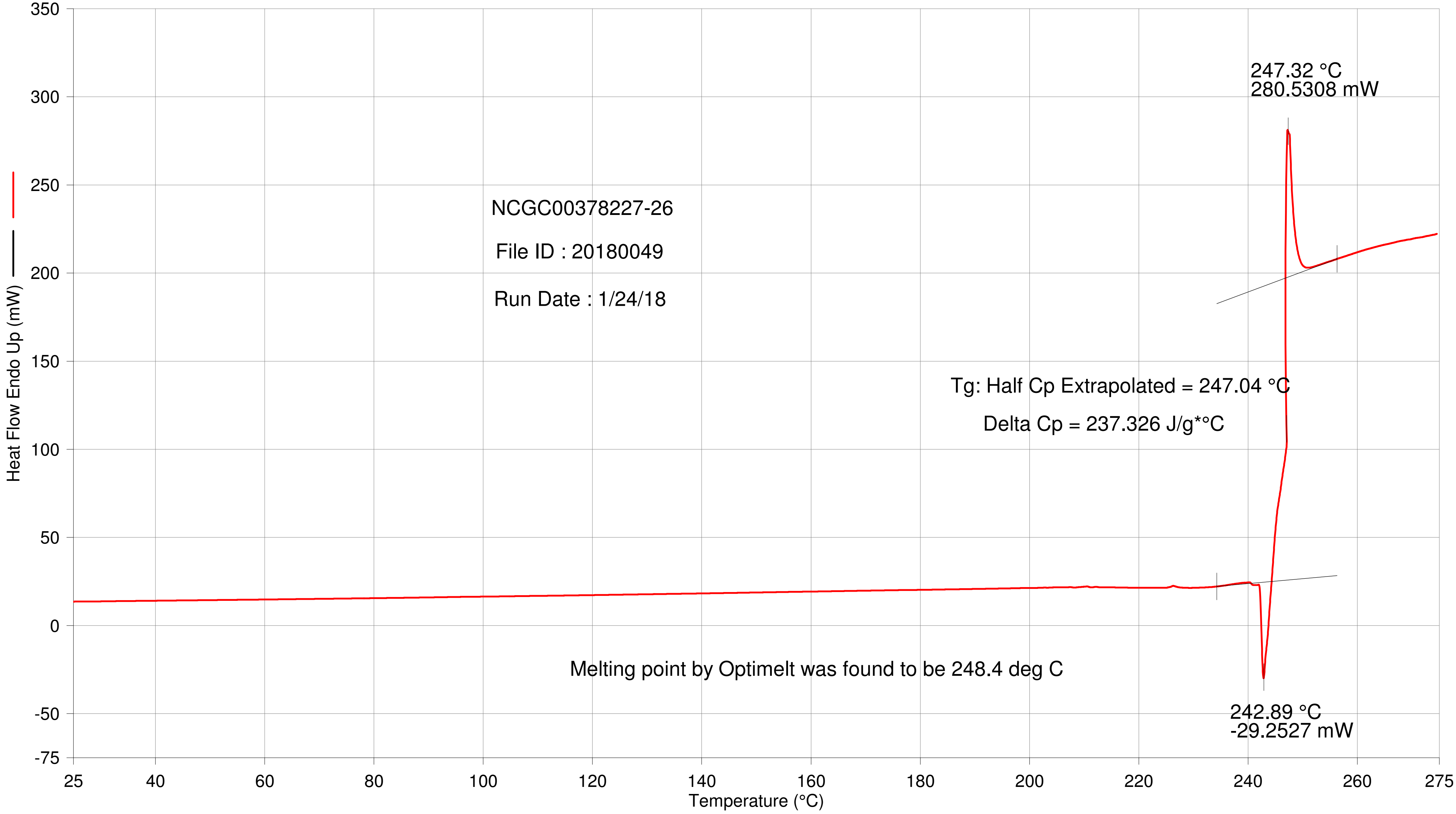

1/25/2018 8:44:33 AM

1) Hold for 1.0 min at 20.00°C   2) Heat from 20.00°C to 275.00°C at 10.00°C/min

Filename:	C:\Program Files\PerkinElme...\20180050.tgd
Operator ID:	D Parikh
Sample ID:	NCGG00378227-26 : 20180050
Sample Weight:	5.696 mg
Comment:	P Morris<br>1-23-18

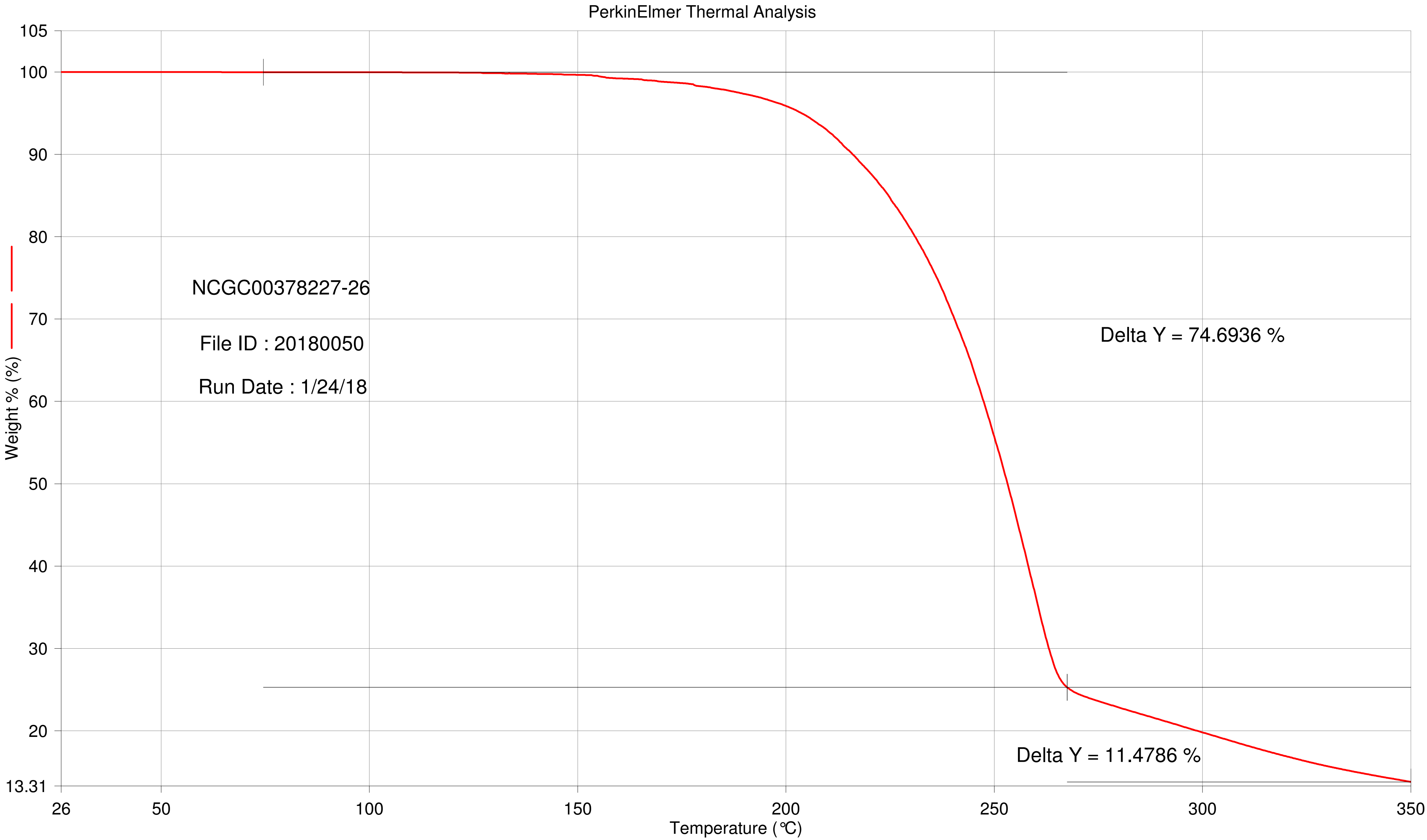

1/25/2018 8:48:08 AM

1) Hold for 1.0 min at 25.00℃	2) Heat from 25.00℃ to 360.00℃ at 10.00℃/min
