# Supplementary material for: Effects of (2R,6R)-hydroxynorketamine in assays of acute pain-stimulated and pain-depressed behaviors in mice
Source: PLoS One. 2024 Apr 19;19(4):e0301848. doi: 10.1371/journal.pone.0301848 (PMC11029659; doi:10.1371/journal.pone.0301848)
Supplement: S2 File — (DOCX) [file pone.0301848.s002.docx]

Hillhouse, Todd (2024). Effects of (2R,6R) HNK on pain stimulated and pain depressed behaviors. figshare. Dataset. <https://doi.org/10.6084/m9.figshare.25289362.v1>
