# Supplementary material for: Effects of (2R,6R)-hydroxynorketamine in assays of acute pain-stimulated and pain-depressed behaviors in mice
Source: PLoS One. 2024 Apr 19;19(4):e0301848. doi: 10.1371/journal.pone.0301848 (PMC11029659; doi:10.1371/journal.pone.0301848)
Supplement: S3 File — (DOCX) [file pone.0301848.s003.docx]

Supplemental Results by Sex

**Figure 1. Effects of sex and acute dosing of ketoprofen or (2*R*,6*R*)-HNK on acid-stimulated writhing and acid-depressed rearing.** (A, B) Treatment with ketoprofen completely reversed the effects of acid on writhing and rearing. Treatment with 3.2 mg/kg ketoprofen produced a significant main effect of dose for writhing (Figure 3C; F(2, 26) = 24.24, P < 0.001); however, there was no main effect of sex (F(1, 13) = 0.008, NS) and no significant interaction (F(2, 26) = 0.02, NS). Treatment with (2*R*,6*R*)-HNK failed to reverse acid-stimulated writhing (Figure 2A). There was no significant interaction (F(3, 24) = 0.09, NS), main effect of dose (F(3, 24) = 0.79, NS), or a main effect of sex (F(1, 8) = 1.63, NS). For acid-depressed rearing (Figure 2B), there was no significant interaction (F(3, 24) = 0.51, NS), main effect of dose (F(3, 24) = 2.79, P = 0.06), or a main effect of sex (F(1, 8) = 3.56, NS). *P < 0.05, ***P < 0.001 represents a significant reversal versus control groups. +++P < 0.001, represent significant effect versus vehicle + 0.56% acetic acid. All significant ANOVAs were followed by a Tukey post hoc test. All data show mean ± SEM. Panel A, B, (Male N = 4, Female N = 6)

**Figure 2. Effects of sex and acute dosing of (2*R*,6*R*)-HNK on acid-depressed locomotor activity and rearing.** For distance traveled (Figure 2A) ketoprofen produced a significant interaction (F(2, 30) = 3.54, P = 0.04), main effect of dose (F(2, 30) = 32.52, P < 0.001), and a main effect of sex (F(1, 15) = 7.97, P = 0.01). A Tukey post hoc test revealed that female mice had significantly higher locomotor activity as compared to male mice under control conditions (vehicle+DH2O; P < 0.001). Additionally, treatment with 3.2 mg/kg ketoprofen completely reversed acid-depressed locomotor activity in both female (P <0.001) and male (P < 0.01) mice. Ketoprofen (3.2 mg/kg) completely reversed acid-depressed rearing (Figure 2B) regardless of sex. There was a significant main effect of dose (F(2, 30) = 20.56, P < 0.001), no main effect of sex (F(1, 15) = 1.48, NS), and no interaction (F(2, 30) = 0.32, NS). For distance traveled (Figure 2C), there was a significant main effect of acid condition (F(3, 20) = 9.12, P < 0.001), no significant interaction (F(9, 60) = 0.85, NS), and no main effect of dose (F(3, 60) = 1.17, NS). Treatment with 0.56% acetic acid significantly decreased distance traveled (P < 0.001). Treatment with (2R,6R)-HNK failed to reverse acid-depressed locomotor activity (Figure 2C). For rearing (Figure 2D) there was a significant main effect of acid condition (F(3, 20) = 18.11, P < 0.001), no significant interaction (F(9, 60) = 1.38, NS), no main effect of dose (F(3, 60) = 1.91, NS)]. Treatment with 0.56% acetic acid significantly decreased rearing, which was not reversed by (2R,6R)-HNK (P < 0.001). All significant ANOVAs were followed by a Tukey post hoc test. ***P < 0.001 represents a significant effect versus control groups. ++P < 0.05, +++P < 0.001, represent significant reversal versus vehicle + 0.56% acetic acid. All data show mean ± SEM. Panel A, B (Male N = 6, Female N = 6).
