# Supplementary material for: Effects of (2R,6R)-hydroxynorketamine in assays of acute pain-stimulated and pain-depressed behaviors in mice
Source: PLoS One. 2024 Apr 19;19(4):e0301848. doi: 10.1371/journal.pone.0301848 (PMC11029659; doi:10.1371/journal.pone.0301848)
Supplement: Attachment — Submitted filename: Response to Reviewers.docx [file pone.0301848.s004.docx]

**Response to Reviewers**

**Reviewer #1:**

The study demonstrated that (2R,6R)-hydroxynorketamine fails to produce antinociception even when it was applied as three intermittent doses. Since study is contradictory to several earlier findings, it is suggested to check the quality and reliability of the HNK molecules used for this study. Authors to provide the detail of the (2R,6R)-hydroxynorketamine like catalogue number, purity and grade.
It is suggested that at least hot plate test should be carried out using HNK molecule from three different source to rule out the quality issue.

RESPONSE: We have contacted the National Center for Advancing Translational Sciences (NCATS) to confirm the purity of the compounds. They have provided us with a certificate of analysis that we have included in the supplemental materials. Additionally, we have added the following information to the drug sections (Page 6, Lines 120-121) “(2*R*,6*R*)-HNK was provided by the National Center for Advancing Translational Sciences (NCATS; Bethesda, Maryland, USA; purity >99.5%, certification of analysis is available in the supplementary materials).”

Although we understand why the reviewer suggested that we test 3 different sources of HNK to rule out quality issues, we have confirmed the purity and provided the required documentation. It should be noted that NCATS is the chemist group that provides (2R,6R)-HNK to the Gould research group, which has published the most on the antidepressant effects of (2R,6R)-HNK (Zanos et al., 2019). Additionally, we feel this would not be a great use of animals from an ethical standpoint and does not align with ARRIVE or the 3 R’s. Lastly, and most importantly, there is a history of (2R,6R)-HNK producing negative results (no significant effects) (Shirayama et al., 2018; Xiong et al., 2019; Yang et al., 2017; Zhang et al., 2018). In fact, the *British Journal of Pharmacology* invited us to write a commentary on the replication issues of HNK (Hillhouse et al., 2019).

Hillhouse, T., Rice, R., & Porter, J. (2019). What role does the ( 2R,6R )‐hydroxynorketamine metabolite play in the antidepressant‐like and abuse‐related effects of ( R )‐ketamine? *British Journal of Pharmacology, 176*. doi:10.1111/bph.14785

Shirayama, Y., & Hashimoto, K. (2018). Lack of Antidepressant Effects of (2R,6R)-Hydroxynorketamine in a Rat Learned Helplessness Model: Comparison with (R)-Ketamine. *Int J Neuropsychopharmacol, 21*(1), 84-88. doi:10.1093/ijnp/pyx108

Xiong, Z., Fujita, Y., Zhang, K., Pu, Y., Chang, L., Ma, M., . . . Hashimoto, K. (2019). Beneficial effects of (R)-ketamine, but not its metabolite (2R,6R)-hydroxynorketamine, in the depression-like phenotype, inflammatory bone markers, and bone mineral density in a chronic social defeat stress model. *Behavioural Brain Research, 368*, 111904. doi:<https://doi.org/10.1016/j.bbr.2019.111904>

Yang, C., Qu, Y., Abe, M., Nozawa, D., Chaki, S., & Hashimoto, K. (2017). (R)-Ketamine Shows Greater Potency and Longer Lasting Antidepressant Effects Than Its Metabolite (2R,6R)-Hydroxynorketamine. *Biol Psychiatry, 82*(5), e43-e44. doi:10.1016/j.biopsych.2016.12.020

Zhang, K., Fujita, Y., & Hashimoto, K. (2018). Lack of metabolism in (R)-ketamine’s antidepressant actions in a chronic social defeat stress model. *Scientific Reports, 8*(1), 4007. doi:10.1038/s41598-018-22449-9

Zanos, P., Highland, J. N., Stewart, B. W., Georgiou, P., Jenne, C. E., Lovett, J., . . . Gould, T. D. (2019). (2R,6R)-hydroxynorketamine exerts mGlu2 receptor-dependent antidepressant actions. *Proceedings of the National Academy of Sciences, 116*(13), 6441. doi:10.1073/pnas.1819540116

**Reviewer #2:**

Hillhouse et al report a series of studies evaluating the ketamine metabolite (2R,6R)-hydroxynorketamine, for potential antinociceptive properties in response to thermal stimuli (hot plate test) and attenuation of acetic acid withing behavior. They also screen the compound in restore rearing behavior, an operationally defined pain-depressed behavior evoked by the acetic acid administration. The rationale behind the studies is primarily to extend the current literature to include acute pain studies, as the literature predominately reports the efficacy of this compound in rodent models of chronic pain. This rationale is weak given that at least two other published articles have reported antinociception using the hot plate Yost et al 2022, JPET (intraperitoneal administration) and Goswami et al., 2023, CEPP, (intranasal administration) and acute inflammatory pain models, formalin (Goswami 2023) and carrageenan (Yost 2022, Neuropharmacology). However, given the relatively few reports in the literature, the content of the manuscript has merit and should be of interest to the field. Below are comments that the authors should consider addressing.

RESPONSE: To be fully transparent, we started collecting the data for this manuscript in fall 2019, but we had to close the lab due to Covid restrictions in early 2020. At the time we started collecting data, there was only one publication that evaluated the effects of HNK on chronic pain (Kroin et al., 2018). Once Covid restrictions were lifted we started collecting data again, then we found the publications by Lucki’s research group (Yost et al., 2022). Based on the findings in the Yost et al., (2022) article, we added the intermittent dosing data to rule out the pretreatment time/dosing (30 min vs 24 hour) and to expand the findings.

We feel we have provided a rationale for our studies in the introduction. We have added text to further support the need to test HNK in assays of pain-depressed behavior as these provide a translational value that is missing from other studies.

Pages 3-4, Lines 69-95: “To date, (2*R*,6*R*)-HNK has only been evaluated in assays of pain-stimulated behaviors, which exclusively rely on behaviors that increase in rate, intensity, and frequency following the presentation or administration of a noxious stimulus (Hillhouse and Negus, 2016; Negus et al., 2010; Negus, 2019). Pain-stimulated behaviors can be attenuated/reversed by drugs that block or reduce the sensitivity to the noxious stimulus *OR* by drugs that produce motor sedation or impair general locomotor activity (e.g. haloperidol or kappa agonists). Although results from pain-stimulated studies are promising and can provide valuable information about candidate analgesics, complete reliance on assays of pain-stimulated behavior may prove to be problematic leading to false positives (impair motor function as described above). Assays of pain-depressed behaviors can be defined as behaviors that decrease in rate, intensity, or frequency following to administration/presentation of a noxious stimulus and used alone or to complement assays of pain-stimulated behavior (Negus et al., 2010; Negus et al., 2019; Stevenson et al., 2009). These pain-depressed behavioral assays provide a translation aspect that is missing with pain-stimulated behavior, as people dealing with pain typically decrease their activity and work productivity (Kawai et al., 2017). Additionally, pain-depressed assays are able to identify compounds that produce false positives of pain-stimulated due to motor impairment as these compounds would not be able increase behaviors back to baseline levels. In the present study, we used an assays of pain-depressed locomotor activity as both acetic and lactic acid produce a robust depression of locomotor activity that can be reversed with nonsteroidal anti-inflammatory drug (NSAID) as well as standard opioid drugs (Negus et al., 2023; Stevenson et al., 2009). In addition to determining if a drug can reverse the acid-induced decrease in locomotor activity and rearing, this assay allows us to evaluate the effects of each drug on locomotor activity in the absence of acid. Specifically, the combination of drug + DH2O (drug alone) is used to rule out false positives as an increase in locomotor activity in the absence and presence of the acetic acid indicated a nonselective increase in locomotor activity not an antinociceptive effect, Additionally, a decrease in locomotor activity in the absence and presence of the acetic acid is a nonselective suppression of behavior resulting in a false negative.”

Major concerns
Study Design: While endeavoring to include both sexes, the variability of the dataset has weakened the ability of the authors to detect meaningful changes with such a low n per sex per group. Please provide the power analysis for each the reported studies. The variance is high given the sex differences between the two studies, with more consistent responses between sexes in the ketoprofen study and indeed greater variation between the sexes in the HNK study relative potentially due to the Latin Square Design. Moreover, based on the literature, females exhibit behavioral response in response to higher doses that those required by male mice. Given the variance in the model at baseline in terms of sexes it seems like it is not sufficiently powered here for the lower doses given the variance in responses between the sexes. This would lead to the conclusion that greater numbers are required to equivocally assess the outcomes.

**RESPONSE:** We completed a sex and temperature/acid concentration curve for all experiments (Hot plate, fig 1a; writhing, fig3a; locomotor activity/rearing, fig 4a,b) and did not find any significant sex differences under baseline conditions. Additionally, the sex data for the HNK studies all trend in the same direction. Based on these results and the comment above, we have decided to move all of the sex effect graphs (from original submission) to the supplementary materials. We provided the results (statistical values) in the supplementary materials to allow researchers to examine that data as needed.

The data have been reanalyzed using a one-way repeated measures ANOVA with the male and female data combined together. Significant ANOVAs were followed by Tukey multiple comparison tests. It is important to note that this change in analysis did not change the overall outcome of the studies or manuscript.

Acetic Acid Writhing: Were those animals used or the ketoprofen study only exposed to acetic acid once?
The use of the Latin square design for (2R,6R)-hydroxynorketamine dose response curve with the acetic acid writhing test. While I appreciated the use of the design to limit the number of animals required, it is questionable for this assay. It is unclear whether the same animals were tested weekly for the acetic acid writhing test or whether the (2R,6R)-HNK doses were administered within a specific timeframe following the first acetic acid writhing induction paradigm. If acetic acid was administered repeatedly, this is no longer an acute inflammatory insult with repeated inductions. Did you observe sensitization to the acetic acid in these animals and hence induce a more chronic inflammatory condition, or greater pain related responses in either sex?

**RESPONSE:** We apologized for any confusion in the design. We used a within-subject design for all of the studies (expect the intermittent dosing studies). We separated acid administration by one week to ensure tolerance or sensitization did not develop. This is a common method used by the Negus research group (Hillhouse and Negus, 2016; Kwilasz et al., 2012; Kwilasz et al., 2014; Pereira Do Carmo et al., 2009; Leitl et al., 2014; Negus et al., 2010). Additionally, the Negus research group has shown that daily acid administration does not produce tolerance or sensitization (Legakis et al., Miller et al., 2015). We monitor mice daily for any pain and distress for mice receiving once a week acid administration, and did not see any noticeable change in behavior. Additionally, we monitor mice for any exaggerated or lack of response to acid on test days and did not see a change in acid effects.

We have edited the text in the writhing and pain-depressed locomotor activity section to clarify when and how drugs and acid was administered. (Page 8, Lines 163-170) “For the drug experiments, (2R,6R)-HNK (0-32 mg/kg) and the positive control ketoprofen (0 or 3.2 mg/kg) were administered 30 mins prior to 0.56% acetic acid administration (which was selected based on the results from the acetic acid concentration curve experiment). Drug doses were randomized using a Latin-square design. There was a minimum of 48 hours between test sessions, and acetic acid administration was separated by a week. For example, on Monday mice were injected with a drug + 0.56% acetic acid, and on Thursday mice were injected with a drug + DH2O. Separate groups of mice were used for (2R,6R)-HNK (Male N = 4; Female N = 6) and ketoprofen (Male N = 8; Female N = 7).

(Page 9, Line 185-193)” For the drug experiments, (2R,6R)-HNK (0-32 mg/kg) and ketoprofen (0 or 3.2 mg/kg) were administered 30 mins prior to 0.56% acetic acid or vehicle (deionized water) injections. Acid vehicle tests were used to determine if (2R,6R)-HNK or ketoprofen could reverse acetic acid depression of LMA without stimulating locomotor activity. Drug doses were randomized using a Latin-square design. To be consistent with the writhing experiments, there was a minimum of 48 hours between test sessions, and acetic acid administration was separated by a week. For example, on Monday mice were injected with a drug + 0.56% acetic acid, and on Thursday mice were injected with a drug + DH2O. Separate groups of mice were used for (2R,6R)-HNK (Male N = 9; Female N = 8) and ketoprofen (Male N = 6; Female N = 6).

In addition, the logistics of the “reversal test” do not allow for post writhing induction treatment. All of these evaluations are prophylactic studies.

**RESPONSE:** We have removed the phrase “For the drug reversal experiments” (line 163 and 185) and replaced with “For the drug experiments”. The quotes in the previous response show that “reversal” was removed.

Hot plate Assay: Please confirm whether “first paw lick” refers to the hindpaws, forepaws or any paw? Did you have a different behavioral profile of thermal responses in male and female mice? Did the raters always agree on the first response?

**RESPONSE:** We have edited the text to state “first forepaw or hindpaw lick”. Figure 1a shows the effects differences for the hot plate temperature-curve. We found no sex differences in the temperature-curve OR on baseline values for the animals treated with drug (Figure 1b and 1c). Our raters were very reliable. In the rare event that both raters did not stop their stopwatch at the same time, the experiment continued until the second rater saw a behavioral response (paw lick, paw shake, or jump). Using the average time of the two raters for all mice help account for any differences in rater’s times. We found this to be a better option than relying on a single rater.

Time of testing post treatment: In the reported literature, testing time points post treatment for intraperitoneal dosing indicate the onset of drug effects analgesia and antinociception as occurring 2-4 hours post treatment, with the optimal effects observed at 24 h post treatment. A similar profile for intrathecal administration has been reported, with intranasal (2R,6R)-HNK resulting in more rapid activity, with significant antinociception on the hot plate at 30- and 60-minutes following treatment. Similarly, for the intermittent dosing study, the authors reference Wulf et al., which again examined the efficacy of (2R<6R)-HNK at 24 h following the final treatment. Thus, the time points of testing used in this manuscript (30 minutes post treatment) are potentially to early to yield significant effects.

**RESPONSE:** As mentioned above, a significant portion of the data reported in our manuscript were collected prior to the publications that provide the 24hr timepoint. Intranasal HNK administration produce antinociceptive effects at all time points test (15, 30, and 60 mins). I would like to point out that these effects were very small (increase of 5 sec at the max effect) with an N = 10.

We agree that the pretreatment time could be playing a role which is why it is discussed in the discussion (Lines 320-330). Additionally, we added the intermittent dosing studies to address the issue. The mice received 4 injections over 6 days (injections on days 1, 3, 5, and the last injection was 30 mins before the test on day 6). This dosing regimen produced antidepressant effects in Wulf et al., 2022, but failed to produce an antinociception response in hot plate and pain-depressed locomotor activity. There was an injection 24 hours before the test, so this experiment helps rule out the pretreatment time issue.

While we were collecting the 56-degree hot plate data, we noticed that there was no effect of (2R, 6R) HNK so we decided to collect a 24-hour time point (24 hours after the final cumulative dose). We did not include this these data in the original submission as (2R,6R) HNK failed to produce an antinociceptive response at 24 hours (it actually decreased withdrawal latency), and we did not feel it added much to the manuscript. However, the reviewer has raised concerns about the time point, so we have decided to include the 24-hour time point data for the 56-degree hot plate. Please find the 24-hour time point data in figure 2.

Statistical Analysis: Please do not include Tukey multiple comparison test when there were no significant interactions. If you do wish to look for simple main effects, then you need to modify your post hoc test and do not use a Tukey comparison.
Why not just run a three-way ANOVA to evaluate Sex X Experimental condition X Dose? Or indeed a repeated two way ANOVA for the hot plate studies.

**RESPONSE:** Based on the comments above sex, we have decided to move all of the sex effects graphs (from original submission) to the supplementary materials. The data has been reanalyzed using a one-way repeated measures ANOVA with the male and female data combined together. Significant ANOVAs were followed by Tukey multiple comparison tests.

We have added the following text to the Data Analysis section (Lines 212-216) “As no significant sex effects were found in the baseline control experiments OR in the (2R,6R)-HNK experiments, we reanalyzed the data using a one-way repeated measures ANOVA without sex as a factor. For transparency purposes, figures and analysis that used sex as a factor are available in the supplemental materials (these figures align with figures 1, 2, and 3 in the main article).”

Studies not referenced: The authors should look at the following articles for completeness
Goswami N, Aleem M, Manda K. Intranasal (2R, 6R)-hydroxynorketamine for acute pain: Behavioural and neurophysiological safety analysis in mice. Clin Exp Pharmacol Physiol. 2023; 50(2): 169-177. doi:10.1111/1440-1681.13737
Liu, AR., Lin, ZJ., Wei, M. et al. The potent analgesia of intrathecal 2R, 6R-HNK via TRPA1 inhibition in LF-PENS-induced chronic primary pain model. J Headache Pain 24, 141 (2023). [https://doi.org/10.1186/s10194-023-01667-1](https://nam10.safelinks.protection.outlook.com/?url=https%3A%2F%2Fdoi.org%2F10.1186%2Fs10194-023-01667-1&data=05%7C02%7Chillhout%40uwgb.edu%7C194dd83d38d64c5186c208dc16c4e27c%7C7fc34f9d1f754f96b5b33cdcaab03aea%7C0%7C0%7C638410283308070197%7CUnknown%7CTWFpbGZsb3d8eyJWIjoiMC4wLjAwMDAiLCJQIjoiV2luMzIiLCJBTiI6Ik1haWwiLCJXVCI6Mn0%3D%7C3000%7C%7C%7C&sdata=x8zR2DiC8ZAVe%2BsE90HFF6oS1%2FxAOCMIfRKwZOB%2BNWQ%3D&reserved=0)

**RESPONSE:** We have added these citations to the introduction (lines 65-67) and discussion (321-323).

Graphical representation of data:
For all of the study designs and the figures, please indicate the time post treatment at which the drugs were evaluated.
Figures are quite pixelated graphs.
Figure 2 - the word (2R,6R)-HNK in the middle of the graph is not type set correct.
The data representation does not always match the data analysis performed. The overall presentation is quite confusing, particularly for the acetic acid writhing and rearing tests.

**RESPONSE:** We have made the corrections throughout the manuscript.

Minor comments:
The use of the term “pain-stimulated” is perhaps not the quite right. For the hot plate test this is really a noxious thermal stimulus, or simply a test of nociception rather than pain per se, which is a very human based concept. Acetic acid writhing is again a behavioral model that relies on an inflammatory response and the activation of nociceptors. It is always difficult to determine the most meaningful phrasing without over reaching and using terms that are not quite accurate for the conditions under evaluation.

**RESPONSE:** We define “Pain-stimulated” and “Pain-depressed” behavior in the introduction (Page 3-4, Lines 69-95) with citations from published literature. The definition provided states “presentation or administration of a noxious stimulus” will increase (pain-stimulated) or decrease (pain-depressed) behavior. We use the term noxious stimulus as this could be acid or thermal. We use these terms to identify if the noxious stimulus (heat or acid) is increasing or decreasing behavior. We feel there is enough data in the literature to support this language.

The validity of rearing in the open field as a pain depressed behavior is somewhat controversial. Is this truly a pain depressed behavior as we know it in the context of human pain. It would be more useful to have some active engagement rather than passive exploration in the open field. This is perhaps more relevant in those chronic rodent models, where the use of limb is downgrading to prevent pain, or indeed that motor activity is reduced to limit the associated “pain”. There are other models that do require functional activities, nesting, food gathering, or step-down tests/incline test etc that also better explore functionality. Moreover, I would find it difficult to support a medication that induces hyperactivity rather than a restoration of normal function.

Is it more beneficial to have a compound that produces overall anti-nociception rather than just analgesia alone. I would not be happy with a compound that modulated this behavior.

**RESPONSE:** We thank the reviewer for sharing their concerns regarding the rearing outcome of the pain-depressed locomotor activity. However, we would like to point out that rearing behavior was decreased in an acid-concentration dependent manner that was consistent with the decrease in locomotor activity (Figure 3A, B). Additionally, treatment of ketoprofen completely reversed rearing behavior back baseline rearing levels. Taken together, these data support that the noxious stimuli acetic acid is responsible for the decrease in rearing and it is reversed by an NSAID. We feel it is important to include these data in the manuscript as it further supports the lack of HNK effects and does not add much length to the manuscript.

We agree with the reviewer that we do not want a medication that induced hyperactivity. The goal of pain-depressed locomotor activity is to find a dose of a drug that does not effect locomotor activity in the absence of acid while restoring acid-induced depression of locomotor. We have expanded the introduction to better explain our rationale for using pain-depressed locomotor activity and it directly addresses this concern.

Page 4, Lines 83-95: “Additionally, pain-depressed assays are able to identify compounds that produce false positives of pain-stimulated due to motor impairment as these compounds would not be able increase behaviors back to baseline levels. In the present study, we used an assays of pain-depressed locomotor activity as both acetic and lactic acid produce a robust depression of locomotor activity that can be reversed with nonsteroidal anti-inflammatory drug (NSAID) as well as standard opioid drugs (Negus et al., 2023; Stevenson et al., 2009). In addition to determining if a drug can reverse the acid-induced decrease in locomotor activity and rearing, this assay allows us to evaluate the effects of each drug on locomotor activity in the absence of acid. Specifically, the combination of drug + DH2O (drug alone) is used to rule out false positives as an increase in locomotor activity in the absence and presence of the acetic acid indicated a nonselective increase in locomotor activity not an antinociceptive effect, Additionally, a decrease in locomotor activity in the absence and presence of the acetic acid is a nonselective suppression of behavior resulting in a false negative.”

In the discussion when referring to the differences between the current and previously published methods, please also discuss the differences in apparatus for the hot plates used and the level of subjectivity to these assays. Now as more reports are emerging that use more automated recording of responses this should improve with reports. In addition, the intranasal study dose shows significant antinociception on the hot plate within the hour of administration - again with a much higher n that included here for one sex only.

**RESPONSE:** We have added the requested text (Lines 321-330). “Interestingly, Goswami and colleagues (2023) found that intranasal administration of (2R, 6R)-HNK produces a more rapid antinociceptive response in the 52ºC hot plate assay. We failed to find antinociceptive effects in the 52ºC hot plate with intermittent dosing when (2R,6R)-HNK had been on board for at least 24 hours (4 injections over 6 days). It is also important to note that there is a level of subjectivity in the hot plate assays as experimenters are tasked with identifying behaviors, which can be subtle, from a distance to not disrupt the mouse’s behavior. The hot plate is not an automated assay, which leaves room for experimenter error. Additionally, it is possible that the size of the hot plate influence behavior as a larger plate allows the mouse more space to move providing competing behavior (thermal response [lick, shake or jump] vs walking/exploring).”

The intranasal studied used an N = 10 (males only). As stated earlier, we have decided to move the sex effect data (drafts from first submission) to the supplementary materials. Now we have combined the male and female data (N = 12) to increase our power. This sample size (N = 12, male = 6, female = 6) is standard in the field now.
