# Supplementary material for: Effects of (2R,6R)-hydroxynorketamine in assays of acute pain-stimulated and pain-depressed behaviors in mice
Source: PLoS One. 2024 Apr 19;19(4):e0301848. doi: 10.1371/journal.pone.0301848 (PMC11029659; doi:10.1371/journal.pone.0301848)
Supplement: Attachment — Submitted filename: Response to Reviewers - PONE-D-23-36070R1.docx [file pone.0301848.s005.docx]

Reviewer #1: Several studies by Jaclyn N. Highland and his team, including those with a comprehensive pharmacokinetics and pharmacodynamics of the HNKs are available suggesting the beneficial effect of HNK. Therefore, current contradictory finding is justifiable only if sufficient data has to be presented to rebut it mechanistically. If not, at least thermal nociception has to be assessed for HNK from three independent sources.

REPONSE: We based our pretreatment times on the bioavailability data provided in the Highland et al 2019 paper. Highland and colleagues found that HNK peaked at 15 mins and was still present in brain and plasma at 30 mins. HNK was undetectable after 1 hour. Therefore, we decided to use a 30 min pretreatment based on brain and plasma concentration. This is discussed on Page 16-17 lines 360-375 and Highland et al 2019 is also cited in the Drugs and Chemicals Section.

We understand the reviewer’s point that other studies have found antinociceptive effects of HNK in different assays. Most of these positive effects are found in chronic pain models rather than acute pain models, which is discussed in the introduction as well as the discussion. However, this is NOT the first time that independent research has failed to replicate or find positive effects of HNK – this was noted in our last response to reviewers.

To compromise with Reviewer #1 we have decided to change the name of our manuscript slightly. The previous title was “(2R,6R)-hydroxynorketamine fails to produce antinociception in assays of acute pain-stimulated and pain-depressed behaviors in mice.” The new title is “Effects of (2R,6R)-hydroxynorketamine in assays of acute pain-stimulated and pain-depressed behaviors in mice”

Additionally, we added an “Other Considerations” paragraph in the discussion (pages 17-18, lines 378-398) to discuss the variables that could be considered confounding variables (this is in response to the reviewers last comment).

In the current study, authors have to revisit their assays of behavioral phenotyping and the quality of HNK. The quality certificate provided by authors is indicative of “quality at source” but not the “quality at bench”.

RESPONSE: We disagree with the Reviewer #1 on the statement that “the authors have to revisit their assays of behavioral phenotyping.” We have provided baseline tests with sex as a factor for ALL behavioral assays. Additionally, we have shown that the positive control morphine produced the appropriate antinociception response in all hot plate tests (figure 1). We also demonstrated that the positive control ketoprofen reversed acid-induce writhing (Figure 3) and pain-depressed locomotor activity (figure 4). Collectively, these data support that there are no issues with our behavioral assays or procedures.

We have provided the certificate showing that HNK was pure. There is absolutely no reason to believe that the quality of HNK changed in our hands. We stored HNK per the manufacturer directions. Additionally, and most importantly, we had significant effects of HNK in the 56C hot plate at 30 mins (Fig 1C) and 24 hours (fig2) as well as a significant increase in rearing (fig 3F) during the acid-induced writhing assays (there was also a trend towards a reduction in writhing at 10 mg/kg; fig3E). Taken together, these data prove that HNK was active “at the bench”.

Please also check a technically robust paper on even the oral bioavailability of HNK in Mouse, rat, and dog . Highland, Jaclyn N., et al. "Mouse, rat, and dog bioavailability and mouse oral antidepressant efficacy of (2R, 6R)-hydroxynorketamine." Journal of Psychopharmacology 33.1 (2019): 12-24.

**RESPONSE:** We know this paper and based the pretreatment time of our studies on the bioavailability in mouse brain and plasma (we have added the citation to our drug section). Highland and colleagues found that HNK peaked at 15 mins and was still present in brain and plasma at 30 mins. HNK was undetectable after 1 hour. Therefore, we decided to use a 30 min pretreatment based on brain and plasma concentration. This is discussed on Page 16-17 lines 360-375.

Another study published in the FASEB journal suggest the Analgesic and Antinociceptive Effects of (2R,6R)-hydroxynorketamine (HNK) in Mice even after 24 hrs of drug administration([https://doi.org/10.1096/fasebj.2022.36.S1.R2181](https://nam10.safelinks.protection.outlook.com/?url=https%3A%2F%2Fdoi.org%2F10.1096%2Ffasebj.2022.36.S1.R2181&data=05%7C02%7Chillhout%40uwgb.edu%7Ca6bc2ad096ed46696ea708dc3d83822e%7C7fc34f9d1f754f96b5b33cdcaab03aea%7C0%7C0%7C638452882270697940%7CUnknown%7CTWFpbGZsb3d8eyJWIjoiMC4wLjAwMDAiLCJQIjoiV2luMzIiLCJBTiI6Ik1haWwiLCJXVCI6Mn0%3D%7C0%7C%7C%7C&sdata=5UEBL%2F9AbwTRE2XfIRbGOTdCwIMuuiarVdBEiGPOwDo%3D&reserved=0) )

**RESPONSE**: As stated in the previous revision, we collected most of these data prior to the Yost publication. It is important to note that Yost, only tested one acute assay (50C hot plate) – The other assays were for a chronic pain, which is very different. Yost et al found that HNK only produced an antinociceptive response at 24 hours (not at 10 mins, 1, 4, or 48 hrs). Based on the results of these studies, we added intermittent dosing studies that addressed the criticism of the 30 min pretreatment time as mice received a total of 4 HNK injections over 6 days. We have added a timeline of injections to figure 5 as there appears to be confusion on the 24 hour pretreatment time. We clearly tested HNK after 24 hours in 3 experiments (56C hot plate [fig 2], intermittent dosing 52C hot plate [fig 5] and intermittent dosing pain depressed behavior [fig 5]). We discuss the differences of the present paper and the Yost paper (mentioned above) at length on page 15 Lines 322-339.

Behavioral phenotypes of rodents may also be affected due to several technical, state and trait factors.
In the current study, both the male and female mice were used for assays. The presence of the opposite gender mice in the same housing, test arena and the odor/scent marks of opposite gender is sufficient to perturb the expected behavioral response. Therefore, it is suggested to describe the procedure employed to rule out such confounding factors. Such perturbations are more likely if neighboring mice are in estrous stage.
Following relevant paper of Nature Neuroscience is an example to explain that how important are the state and trait factors in pre-clinical behavioral pharmacology.
Georgiou, Polymnia, et al. "Experimenters’ sex modulates mouse behaviors and neural responses to ketamine via corticotropin releasing factor." Nature neuroscience 25.9 (2022): 1191-1200.

RESPONSE: All behavioral apparatus were cleaned with 50% EtOH to remove odor/scent marks. We have added a statement to the methods section for each assays.

We have added a paragraph in the discussion (Page 17-18 Lines 378-398) to discuss the variables mentioned above.

Reviewer #2: Thank you for addressing the comments and including the additional data. Overall, the manuscript is a very interesting report and important for the field. There remain some edits that should be considered for clarity.

With regards to the statistical analysis, following a One -way ANOVA for this data set it is more appropriate to utilize a Dunnett multiple comparison test, with comparison made only to the control group 0 mg/kg rather than exploring differences between all of the doses used. Please make this change.

**RESPONSE:** We have now used Dunnett’s *post hoc* tests to compare the HNK writing data. This did not change any of the statistical results or the conclusions.

Figure 1, 3c - the legend indicating male has an error that needs to be corrected.

**RESPONSE:** Thank for catching this issue. It has been corrected.

It remains difficult to compare the ketoprofen data with that of HNK as figure 4e and 4f are presented differently to 4 c and 4d, I realize this is due to the dose response curve but it would make the point more salient if you could graph this as histograms as per 4c and 4 d

**RESPONSE:** We have made edits to the ketoprofen and HNK figures in figure 3 and 4 to make them as similar as possible for comparisons.

In addition, I'm not sure whether it is appropriate to separate the ketoprofen data by sex when you have decided to collapse the data across the sexes for the HNK data. However, it is apparent that there is a main effect of sex on withdrawal latencies on the hot plate at 56C. Given the minor sex differences noted above, it might be worthwhile maintaining your original figures with sexes identified.

**RESPONSE:** For the main manuscript, we have re-graphed the ketoprofen data to be consistent with the HNK data (see data has been moved to the supplemental material). We have added back in the sex data for the hot plate assay since there was an HNK mediated sex differences in the 56-degree hot plate.
